# Advanced rehabilitation technology in orthopaedics—a narrative review

**DOI:** 10.1007/s00264-020-04814-4

**Published:** 2020-10-13

**Authors:** Yuichi Kuroda, Matthew Young, Haitham Shoman, Anuj Punnoose, Alan R. Norrish, Vikas Khanduja

**Affiliations:** 1 Young Adult Hip Service, Department of Trauma and Orthopaedic Surgery, Addenbrooke’s-Cambridge University Hospitals NHS Foundation Trust, Hills Road, Box 37, Cambridge, CB2 0QQ UK; 2 Department of Academic Orthopaedics, Trauma and Sports Medicine, Queens Medical Centre, University of Nottingham, Nottingham, UK

**Keywords:** Rehabilitation, Technology, Orthopaedics, Telerehabilitation

## Abstract

**Introduction:**

As the demand for rehabilitation in orthopaedics increases, so too has the development in advanced rehabilitation technology. However, to date, there are no review papers outlining the broad scope of advanced rehabilitation technology used within the orthopaedic population. The aim of this study is to identify, describe and summarise the evidence for efficacy for all advanced rehabilitation technologies applicable to orthopaedic practice.

**Methods:**

The relevant literature describing the use of advanced rehabilitation technology in orthopaedics was identified from appropriate electronic databases (PubMed and EMBASE) and a narrative review undertaken.

**Results:**

Advanced rehabilitation technologies were classified into two groups: hospital-based and home-based rehabilitation. In the hospital-based technology group, we describe the use of continuous passive motion and robotic devices (after spinal cord injury) and their effect on improving clinical outcomes. We also report on the use of electromagnetic sensor technology for measuring kinematics of upper and lower limbs during rehabilitation. In the home-based technology group, we describe the use of inertial sensors, smartphones, software applications and commercial game hardware that are relatively inexpensive, user-friendly and widely available. We outline the evidence for videoconferencing for promoting knowledge and motivation for rehabilitation as well as the emerging role of virtual reality.

**Conclusions:**

The use of advanced rehabilitation technology in orthopaedics is promising and evidence for its efficacy is generally supportive.

## Introduction

With an ageing population, the number of patients affected by orthopaedic conditions that either require rehabilitation alone or surgical intervention and subsequent rehabilitation are on the rise [1, 2]. As the demand for rehabilitation increases, advanced rehabilitation technology has also developed, but its role and efficacy are not always appreciated by surgeons or therapists. Rehabilitation in orthopaedics often aims to improve range of motion (ROM) and muscle strength around joints. Devices with sensors [3] or robotic technology [4] that enable quantitative measurements of these parameters in three dimensions have been designed and applied in the clinical setting. Feedback and patient self-monitoring have been strongly associated with improved outcomes [5], and these technologies make monitoring and feedback more accessible.

Some advanced rehabilitation technology is bulky, costly and complex and mainly useful for rehabilitation in hospital. However, rehabilitation after many surgical interventions takes place in a patient’s own home [6]. Adherence to home musculoskeletal rehabilitation protocols has been shown to be poor with some studies reporting rate of non-compliance to be 50–65% [7]. With the accessibility of high-speed Internet, teleconferencing has been made possible [8] improving contact in orthopaedic rehabilitation and potentially improving compliance. The use of gaming consoles and other widely available gaming hardware [9] to interact with patients may also improve compliance. In addition, use of this technology may aid therapists to diagnose, treat, and monitor patients’ progress remotely. The therapist may then ensure correct performance of exercises and increase motivation resulting in improved patient adherence [10]. Use of advanced rehabilitation technology in this way enhances home-based rehabilitation and may be a time and cost-efficient alternative to conventional clinical or home-based face-to-face sessions.

Although there has been growing interest in the use of advanced rehabilitation technologies in orthopaedics, an initial scoping review of the literature failed to identify a paper offering a broad overview of this technology used within an orthopaedic population. Therefore, the purpose of this narrative review is to explore the broad variety of technologies that are currently being used in orthopaedic rehabilitation and determine the extent to which these technologies can support and complement traditional services such as physiotherapy.

## Materials and methods

A literature search of journal articles using the PubMed (MEDLINE) and EMBASE databases was conducted in September 2019. No date restrictions were placed. Relevant literature describing rehabilitation technology utilized in orthopaedics was identified from the above appropriate clinical databases and a narrative review was undertaken.

## Rehabilitation technology

This review paper considers advanced rehabilitation technology into two sections: hospital-based rehabilitation and home-based rehabilitation (see Table 1 for summary). It describes each advanced technology and details the current evidence for its use.

**Table 1:** Summary of advanced rehabilitative technologies. *TKR*, total knee replacement; *3D*, 3-dimensional; *ROM*, range of movement

Primary location of use	Technology	Features
Hospital-based technologies	Continuous passive motion	Well established after TKR. Has also been tried in the home setting.
	Robotic devices	Support body weight, particularly after spinal cord injury.
	Electromagnetic sensors	Measures 3D kinematics, but cumbersome outside the gait lab.
Home-based technologies	Inertial sensors	Collect 3D motion data. Inexpensive and portable.
	Software applications	Can be used on a smartphone or tablet. Used for measurement of movement and patient feedback.
	Commercial gaming technology	Detects subtle changes in balance, co-ordination and ROM during functional activities.
	Videoconferencing	Allows remote diagnosis, treatment and monitoring.

### Hospital rehabilitation

#### Continuous passive motion (CPM)

The use of CPM in orthopaedic rehabilitation has been around for two decades and is most commonly reported following total knee replacement (TKR) and has been primarily advocated to improve knee flexion recovery [11, 12]. Naylor et al. reported that greater knee ROM at discharge after TKR was a significant predictor of improved ROM after rehabilitation [13]. A recent meta-analysis provided statistically significant, moderate evidence, indicating that CPM reduced pain, restored knee ROM and enhanced functional recovery after TKR regardless of follow-up duration [14]. It has been suggested that CPM leads to positive biological effects on tissue healing, oedema and haemarthrosis [11, 15]. CPM is widely used in hospitals in the post-operative period allowing therapists to streamline their workload and improve their capacity to see other patients [6]. CPM has also been used for the rehabilitation of other knee conditions such as ligament reconstruction surgery in both adults [16] and children [17]. CPM is also being trialled for the treatment of other joints, such as the elbow [18] and shoulder [19], but so far, no advantage to CPM has been definitively reported. Smart, user-friendly programmable machines are becoming the norm [20].

#### Electromagnetic sensors

The electromagnetic tracking system (ETS) is a six degrees-of-freedom measuring device that simultaneously records the three-dimensional (3D) position and orientation of multiple electromagnetic sensors attached to body segments using a transmitter [3, 21, 22]. The reliability of ETS for measuring kinematics (e.g. ROM) of the upper and lower limbs has been reported [3, 22]. Nakagawa et al. demonstrated that it can be used to accurately evaluate 3D kinematics such as single leg squat and stepping [21]. The ETS may also be used to measure kinematic changes in a patient over time, which is useful for assessment of rehabilitation interventions. The disadvantage of ETS is that it is cumbersome and unable to be easily used outside of a laboratory or clinical-based setting. Recently, however, a more clinically friendly device that measures knee kinematics using only two electromagnetic sensors (on the thigh and shank) has been reported [23, 24]. This compact ETS also enables quantitative evaluation of the pivot shift test pre- and post-anterior cruciate ligament (ACL) reconstruction.

#### Robotic devices

Rehabilitation in patients with spinal cord injury (SCI), who have reduced or absent sensorial input and motor output, is challenging [25]. Particularly, the support of the patient’s body weight during rehabilitation leads to physical exhaustion of the therapist [26]. To overcome this, robotic supportive devices have been developed that may reduce the physical demands on the therapist [26, 27]. Several studies have demonstrated that robotic-assisted gait training in SCI patients promotes body compensation and neuroplasticity, leading to improvements in cardiorespiratory, urinary, musculoskeletal, neuronal and somatosensory systems [28–32]. A systematic review evaluating robotic assisted gait for SCI also showed similar results [26].

Recently, portable robots such as the ARGO (ARGO Xtreme Terrain Robotics, ON, Canada), EKSO (EKSO Bionics, CA, USA), Indego (Parker Hannifin Corp., OH, USA), ReWalk (ReWalk Robotics, Inc., MA, USA) and WPAL (Fujita Health University, Aichi, Japan) have also been developed, and can perform gait training not only indoors but also outdoors. Hybrid Assistive Limb (HAL; University of Tsukuba, Ibaraki, Japan) has a hybrid system allowing both a voluntary and an autonomous mode of action to support gait training. HAL uses control algorithms and supporting devices that control each knee and hip separately. A systematic review reported that although HAL had beneficial effects on gait, function and independence in walking, no study provided conclusive data on differences between HAL gait-training compared to the other forms of gait-training [33]. On the other hand, Cheung et al. performed a meta-analysis of randomised controlled trials (RCTs) or quasi-RCTs that compared robotic-assisted lower limb training to a control of other treatment approaches or no treatment in SCI patients [4]. They concluded that the robotic-assisted training group showed better improvement in walking independence and endurance than other training methods.

The effect of robotic devices on upper limb rehabilitation in SCI patients is less established. Zarffa et al. reported no difference in the improvement in functional scores for patients receiving Armeo Spring® (Hocoma AG, Switzerland) training on one arm compared to the arm that did not have robotic-assisted rehabilitation, both at discharge and follow-up assessment [34]. To date, only a few studies have evaluated the effects of upper limb robot training after SCI and the evidence for their use is not robust. However, the use of upper extremity robotic rehabilitation devices in persons with SCI has gained increasing research interest, with aims to achieve enhanced functional ability via promotion of neuroplasticity [35].

Koller-Hodac et al. introduced a robot-assisted knee device that can be used at home to acquire a full ROM and joint flexibility after knee surgery [1]. A narrative review by Sicuri et al. suggested that it was reasonable to consider robotic rehabilitation of the shoulder for instability, stiffness, joint replacement, rotator cuff tear or other tendon ruptures [36]. A single-joint training robot, NeXOS (The Nexus Project, University of Abertay Dundee, UK; Bradley et al. [37]) was designed as a prototype to aid individualised lower limb rehabilitation, allowing progression from passive motion, through to active-assisted and on to resistance training. The automated system was also designed to record the patient’s movement, enabling the therapist to comprehensively analyse the effectiveness of exercises and adjust these as required for optimal joint recovery. Various other single joint training robots have been designed [38], but there are few clinical outcome studies to guide specific recommendations for their use in the clinical setting.

Robotic devices face a number of challenges including the pressure of the suit in certain areas, skin irritation, training-related pain, excessive energy consumption due to device use and high costs [26, 33]. Using robotic devices for rehabilitation after SCI may improve motor learning and promote neuroplasticity, possibly reducing secondary complications. This is a very active area of research and development, and it is likely that we will continue to see rapid growth in this field.

### Home-based rehabilitation

#### Inertial sensors

Inertial sensors such as accelerometers, magnetometers and gyroscopes collect 3D motion data, which they communicate to a computer for analysis by accompanying software. This allows them to be used to accurately measure and assess the movement of a patient’s joint during a variety of functional exercises [39]. Saber-Sheikh et al. compared an inertial sensor system (utilising a combination of accelerometers, magnetometers and gyroscopes) to an ETS system to determine their relative abilities to evaluate functional activities such as walking [40]. The accuracy of the two methods were comparable. However, the inertial sensor system had the advantage that it was relatively inexpensive, portable and user-friendly. The inertial sensor system can feasibly be used in a patient’s home or workplace, rather than its use being confined to the laboratory or clinical environment. Kumar et al. demonstrated comparable ROM measurements between a wireless wearable automated inertial sensory system and traditional goniometry [41]. They proposed that the measurements recorded by the sensor system were more reliable, as they were not subject to the variability or subjectivity of practitioner’s use of the goniometer. The combination of both symptomatic and asymptomatic participants and variety of upper and lower extremity applications of the inertial sensors in their series demonstrates that inertial sensor technology is an effective method of quantifying dynamic ROM. However, Tulipani et al. suggested that the inertial sensor system overestimated lumbar flexion compared to the motion camera system [42]. This loss of accuracy may be due factors such as position of sensor placement, reliability of skin attachment or an interaction effect with the sensors. This underlines the importance of standardisation of sensor placement with reliable skin attachment skills together with improved inertial sensor algorithms to optimise the accuracy of the system. The studies cited above show that inertial sensors are useful to assess the impact of treatment modalities, but do not demonstrate their use which delivers improved outcomes.

#### Application software (Apps)

There are many medical Apps available, with some offering knowledge of medical conditions, anatomy, drug information or other treatments [43]. The reliability and validity of Smartphone Apps for measuring ROM or position sense in different joints has been explored in several studies including the cervical spine [44], lumbar spine [45], shoulder [46], elbow [47], knee [48] and ankle [49]. In addition to static ROM evaluation, smartphone-based accelerometers have been deemed to be an equally valid way of measuring dynamic knee ROM compared with a laboratory-based isokinetic dynamometer [50]. Hoshino et al. created an iPad (Apple Inc., Cupertino, CA) App that can process video images and provide data on the translation of the lateral compartment of knee in almost real time [51]. They demonstrated the potential of this App to classify the pivot shift in ACL-deficient patients [51, 52]. Matera et al. combined the accelerometer with smartphone global positioning system (GPS) to create an App that is able to detect, measure and record essential movements of the hand and wrist [43] that can automatically be sent to a medical professional at the end of a therapy session. Twenty participants underwent a four week wrist motion rehabilitation using this App after wrist surgery, which included plate fixation of distal radius fractures and arthroscopic repairs of triangular fibrocartilage complex. They demonstrated significantly improved ROM in every plane of wrist motion.

Apps are also useful for patient feedback. One technology that provides real-time feedback is a sensor called BandCizer^TM^ (BandCizer Aps, Odense, Denmark) for elastic band exercises. It can quantify contraction time, the number of repetitions performed and the force used to stretch the elastic band by measuring the thickness of the band [53]. BandCizer^TM^ can send data to an iPad, and the BandCizer^TM^ App provides users with real-time feedback on exercises [53, 54]. Rathleff et al. reported that 40 adolescents with patellofemoral pain were randomized to treatment with real-time BandCizer^TM^-iPad feedback on contraction time or not. The App significantly improved compliance to the rehabilitation [55]. As elastic exercise bands are a versatile tool used for a range of upper and lower extremity exercises, BandCizer^TM^ application could be utilised across multiple areas of orthopaedic physiotherapy rehabilitation [56].

However, Stove et al. emphasised that subtle variations exist in the quality of measurements depending on the smartphone manufacturer and also that updates of application software complicate the validity of the findings and may lead to inconsistencies in the assessment of measurement such as ROM [50]. Therefore, before clinical utilisation, the accuracy of each application needs to be evaluated and this should be repeated each time the software is updated.

#### Videoconferencing

Videoconferencing (VC) has the potential to play an important role in the management of orthopaedic patients. VC could allow therapists to diagnose, treat and monitor patients’ progress remotely as telerehabilitation in orthopaedic setting.

Eriksson et al. investigated patients’ experiences of participating in therapy via VC at home for 2 months after shoulder joint replacement [10]. This study reported that the access to the guided exercises via VC and the immediate feedback from the physiotherapist led to better knowledge about the body and the surgery, and also improved motivation for daily exercises. Furthermore, patients saw their therapist as expert and problem-solver in the first phase after the surgery allowing them to transition from being dependent and passive, to being independent and active whilst remaining at home.

Tousignant et al. reported on satisfaction with VC for patients following a RCT post TKR [8]. Although high satisfaction rates were noted with the technology, no significant difference was observed when VC was compared to traditional face-to-face rehabilitation. The same research group conducted semi-structured interviews about patients’ perceptions regarding telerehabilitation services [2] and reported that patients were satisfied with most of the aspects of their experience including access to services, their relationship with their therapist, the exercises program, the technology and the support provided by the technical team.

VC promotes the relationship between patients and therapists, knowledge and motivation for rehabilitation, and may reduce costs [57]. However, a recent systematic review concluded that although VC may be acceptable to patients, the day to day workability of VC in clinical practice from a clinician perspective is not always practical [58].

#### Commercial gaming technology

Commercially available gaming systems (CAGS) can detect subtle changes in balance, coordination, strength and ROM of a joint during functional activities [59]. CAGS may serve as a relatively inexpensive and clinician-friendly tool to assess objective functional measurements of patients. CAGS have been reported to promote motivation for therapeutic activities [60] and are an enjoyable method of encouraging physical exercise [61]. The most commonly described CAGS in the rehabilitation setting are the Nintendo Wii with Balance Board (WBB) (Nintendo, Kyoto, Japan) and the Microsoft Xbox with Kinect (Microsoft, Redmond, WA, USA). They utilise several different technologies including integrated accelerometers, infrared detection and movement tracking. Users can access a number of software applications for balance and motion training. The CAGS can also be used to assess postural control and general quality of motion while performing various movement tasks [59]. These devices have the advantage of being marker-less and portable, able to be used in the outpatient clinic, rehabilitation room and at home [62]. CAGS can use therapeutic training protocols that track a patient’s progress, reporting on objective functional measures during rehabilitation stages.

Many studies have shown Microsoft Xbox with Kinect to have an excellent correlation and reliability when compared to a gold-standard motion detection system such as the Vicon MZ motion analysis system (Kverneland Group UK Ltd, St Helens , UK) [63, 64]. However, the Kinect accuracy is reported to be dependent on movement and user position: e.g., the accuracy decreases when the user is sitting [65]. Wochatz et al. evaluated the reliability and validity of the Kinect in lower extremity rehabilitation exercises and reported that the variability was acceptable for joint angles and joint position during the squat, but not during the lunge [62]. Furthermore, there are problems reported with the accuracy of hand tracking [66]. Notwithstanding these limitations, a systematic review of motor rehabilitation using Kinect demonstrated improvements in clinical outcomes such as balance, posture, sensory information and ROM [66].

Yamada et al. reported that the score of Wii Fit (Nintendo, Kyoto, Japan) and Basic Step game correlated with Dual Task Lag of Timed Up-and-Go in older women, which demonstrated association with a real-life outcome [67]. A RCT study showed that Nintendo Wii Fit program versus the conventional rehabilitation after ACL reconstruction had comparable result for knee strength, balance, proprioception, coordination and response time at 8 and 12 weeks [68]. In another RCT study on TKR patients, the rehabilitation using Wii Fit achieved the same ROM, balance and function as the conventional exercise at discharge [9].

CAGS have the potential to make an important contribution in orthopaedic rehabilitation but their role in this context is still in development. Whilst most studies are favourable, some studies have not shown the effectiveness of CAGS when compared to the gold standard [69]. Strong conclusions regarding the reliability and validity of CAGS in the orthopaedic setting cannot be made at this stage [59].

#### The future

Many advanced rehabilitation technologies relevant to orthopaedics are still in development. Virtual reality (VR) technology, which includes an interactive computer environment or games that appear and feel real may also have a role. In physical rehabilitation, VR can be used to personalise treatment, motivate patients, improve compliance and track progress. Currently, there is a lack of strong evidence to support its use. Clinical trials have assessed VR effectiveness in patients with orthopaedic pathology such as ACL injury, frozen shoulder and chronic neck pain. Most of them used off-the-shelf console games such as Nintendo Wii Fit, making it difficult to differentiate from rehabilitation using CAGS [70].

Electromyography (EMG) muscle stimulation devices have shown significant improvements in outcome for patients after TKR [71]. Currently, compact and wireless EMG stimulation devices such as Myo-Ex (Biometrics Ltd., Newport, UK) can be used and have shown rehabilitation benefits in stroke patients [72].

To date, a number of protocol papers for advance rehabilitation technology RCTs have been published in the orthopaedic field [73–75]. As these studies mature, they will provide the high-level evidence needed, when deciding which of these technologies will be useful in the clinical setting.

## Conclusion

This narrative literature review describes the variety of advanced rehabilitation technologies that are currently used in orthopaedics. It outlines the evidence for the extent to which these technologies can support and complement traditional therapy.

Hospital-based technology, such as robotic devices, is widely used mainly in SCI, and it is expected that they will be utilised for other conditions in the future. The reliability of ETS for measuring kinematics of upper and lower limbs has been reported, but the challenge is to develop compact and user-friendly devices. Unlike hospital-based advanced rehabilitation technology, home-based technologies such as inertial sensor, application, and CAGC are relatively inexpensive and user-friendly, making them more accessible. A number of these modalities have been shown to be effective measurement tools in orthopaedics through accurate quantification of patient physical activities such as dynamic ROM and function. VC, a method of telerehabilitation, is acceptable to most patients, and it promotes the patient-therapist relationship and the patient’s knowledge and motivation for rehabilitation.

The use of advanced rehabilitation technology in orthopaedics shows a lot of promise, particularly to support and complement traditional rehabilitation services, and its use is gaining in popularity. These technologies are dependent on the device accuracy and reliability. There remains a paucity of high-level published evidence as to efficacy. Further research is needed to determine the usability, cost-effectiveness and efficacy of advanced rehabilitation technology in high-quality randomised cohorts of orthopaedic patients.
